# Custom Outlier Detection for Electrical Energy Consumption Data Applied in Case of Demand Response in Block of Buildings

**DOI:** 10.3390/s21092946

**Published:** 2021-04-22

**Authors:** Dacian I. Jurj, Levente Czumbil, Bogdan Bârgăuan, Andrei Ceclan, Alexis Polycarpou, Dan D. Micu

**Affiliations:** 1 Electrical Engineering Department, Technical University of Cluj-Napoca, 400027 Cluj-Napoca, Romania; dacian.jurj@ethm.utcluj.ro (D.I.J.); Levente.Czumbil@ethm.utcluj.ro (L.C.); bogdan.bargauan@servelect.ro (B.B.); Andrei.Ceclan@ethm.utcluj.ro (A.C.); 2 Department of Electrical and Computer Engineering and Informatics, Frederick University, 1036 Nicosia, Cyprus; eng.pa@frederick.ac.cy

**Keywords:** data cleaning, demand response, baseline electricity consumption, outliers, local outlier factor (LOF), interquartile range (IQR), density-based spatial clustering of applications with noise (DBSCAN), public buildings

## Abstract

The aim of this paper is to provide an extended analysis of the outlier detection, using probabilistic and AI techniques, applied in a demo pilot demand response in blocks of buildings project, based on real experiments and energy data collection with detected anomalies. A numerical algorithm was created to differentiate between natural energy peaks and outliers, so as to first apply a data cleaning. Then, a calculation of the impact in the energy baseline for the demand response computation was implemented, with improved precision, as related to other referenced methods and to the original data processing. For the demo pilot project implemented in the Technical University of Cluj-Napoca block of buildings, without the energy baseline data cleaning, in some cases it was impossible to compute the established key performance indicators (peak power reduction, energy savings, cost savings, CO_2_ emissions reduction) or the resulted values were far much higher (>50%) and not realistic. Therefore, in real case business models, it is crucial to use outlier’s removal. In the past years, both companies and academic communities pulled their efforts in generating input that consist in new abstractions, interfaces, approaches for scalability, and crowdsourcing techniques. Quantitative and qualitative methods were created with the scope of error reduction and were covered in multiple surveys and overviews to cope with outlier detection.

## 1. Introduction

Demand response applied in aggregation of block of buildings can provide significant benefits, on one hand to the consumers and prosumers and on the other hand to decrease pressure on the transmission and distribution system operators and share the responsibility and benefits of the generators with the rest of the power chain [1].

A demand response in blocks of buildings demo pilot project had been implemented during the period of 2016–2019 in 12 public buildings of the Technical University of Cluj-Napoca (TUCN), consisting in the effective testing of four different demand response automated and/or manual scenarios. The demand response in blocks of buildings (DR-BoB) project started with selecting the public buildings from four different campus locations of the university, having different power signature profiles and different HVAC (heating, ventilation, and air conditioning) systems. Then, in order to effectively implement the demand response (DR) scenarios, a building energy management system (BEMS) was installed at the blocks of buildings (BoBs) technical site, for an online visualization of the energy data and to record a baseline in the energy use and local renewable energy sources (RES) generation. During the following one-year period (12 months) demand response (DR) testing scenarios were implemented in different time schedules, for different combination of the blocks of buildings (BoBs) involved, using automated or manual control, including voluntary involvement of students, academic and administrative staff [2]. Thus, when evaluation of the achieved results followed, the baseline generation issue was the most significant to be properly solved. The paper focuses on this topic, with detailed key approaches regarding outlier detection, recorded data cleaning and baseline construction. Replication of the proposed approach and methodology can be considered, at least for the demand response effectiveness evaluation, to all range of consumers or prosumers, as the key performance indicators: 1—peak power reduction; 2—energy saving; 3—CO_2_ reduction; 4—cost savings, etc. are easy to be applied on a clearly established baseline [3]. Demand response projects can be a great opportunity in local communities not only for the residential energy users, but also for the large pools of public buildings, belonging to the local authorities (schools), utility companies, chain of commercial buildings [4]. Collecting clean electrical energy data from this type of ‘end users’ is a must in order to achieve efficient results.

Regarding outlier’s detection, from all the data science extensive literature some definitions can be summarized for the ‘most common’ data issues. One definition that stands out is given by Barnett and Lewis [5], defining an outlier as an observation or a set of observations that are inconsistent with the rest of the data. According to Edwin de Jonge and Mark van der Loo, “Outliers do not equal errors. They should be detected, but not necessarily removed. Their inclusion in the analysis is a statistical decision” [6]. It is important to mention, that after a significant understanding of the data, it can be concluded that not all the detected outliers should necessarily to be removed or replaced, they can be a meaningful observation on a long term; as a response for this, many distinct outlier detection methods were developed in the literature [7,8]. More than that, in the process of detecting the anomalous values there is only a snowball’s chance in hell to be able to detect multiple outliers given the masking effect that can occur when the outlier cannot be detected due to the presence of the others [9]. This issue was addressed by using sequentially correction of the anomalies or using to reduce the masking effect [10,11]. In [12] the outlier detection techniques were presented as probabilistic models with parametric and nonparametric approaches, statistical models, and machine learning algorithms with clustering-based and classification-based techniques. In the probabilistic model’s probability distribution functions were proposed to detect anomalous data as the values which have the highest probability to go outside a given threshold. There are two types of probabilistic approaches: (a) parametric, where the data is analyzed with an already known distribution function; and (b) nonparametric, where the data is measured based on a density or distance function, the data set which doesn’t behave like the majority of tested population, is considered outlier [13,14]. In most of the parametric and probabilistic outlier detection methods Gaussian distribution functions and median absolute deviation are used [15]. Parametric models are prone to fail because most of the distributions are univariate and the primary distributions of the observations need to be noticed in advanced. Even if the median and mean methods are calculating the central tendency, they are insensitive to the presence of abnormal values. The median method together with median absolute deviation represent the statistical dispersion of the data set and they are more robust than the mean and standard deviation methods. The “breakdown point” [16] is one of the methods used to determine the insensitivity of median method, this indicator represents the maximum number of affected data that can be in the tested data without changing the final results. There is only one problem to address for the median method and that is when more than half of the parameters are of infinite values [17,18]. Non-parametric methods were used on multidimensional data sets using different clustering techniques as *k*-nearest neighbor [19] and Parzen window [20,21,22]. In addition to the most common nonparametric methods, the following were applied: ranking or scoring data, based on differences and similarities [23], Gaussian mixture models [24], and probabilistic ensemble models using the density based local outlier factor (LOF) with distance based as *k*-nearest neighbor [25]. Even if the probabilistic models are often used, there is a possibility that the probabilities can be unavailable or limited due to low correlations in the data, which is why the qualitative cleaning methods can achieve better results in detecting the correct tuples to clean and to reduce the information loss [26].

Statistical methods like auto regressive moving averages [27,28,29,30] and Linear regression models were proposed in [31] and used for outlier detection even if it is hard to detect polynomial functions in real time [32,33]. In general, most of the statistical methods are based on historical data and used for offline anomaly detection even if some of them were described as heavy online anomaly detection techniques [34,35]. Supervised (classification based) and unsupervised learning (clustering based) machine learning methods were used to identify outliers in fraud, health care, image processing, and networks intrusions [36,37,38,39]. One of the most feasible machine learning methods for detecting outliers in an unsupervised environment are the clustering-based methods such as *k*-means [40] or density-based spatial clustering of applications with noise (DBSCAN) [41]. DBSCAN presents some advantages over *k*-nearest neighbors’ method like automatically adjusting the number of clusters to be computed and the ability to isolate the outliers in individual clusters. Neural networks [42,43,44] and support vector machines [45] were also used for anomalous data detection. Functional dependency thresholding with Bayesian optimization for functional dependencies data cleaning purpose was successfully tested on synthetic and real data [46], relaxed functional dependencies were also detected using an improved discovery algorithm relying on a lattice structured search space with new pruning strategy [47].

There has always been a debate on universality in numerical computation, [48] thus providing an opportunity for the authors to test some of the most common techniques from literature [12] in the context of energy consumption. Still, all the data sets may have their own ‘personal character’ or bias. In [49], a preliminary outlier detection empirical testing was conducted by the authors using probabilistic and statistical and machine learning techniques over the Technical University of Cluj-Napoca’s swimming complex. The study highlighted the possibility that some outlier detection techniques would not be able to differentiate between the natural energy peaks (the custom bias or particularity of data) and abnormal data without an additional support function. Therefore, a more detailed investigation has been carried out for all four of the TUCN’s DR-BoB pilot site locations that present different energy consumption patterns with focus on the proper tuning of the implemented/tested outlier detection techniques and evaluation of their effect on baseline construction.

After a short summary of the demand response in blocks of buildings (DR-BoB) project, implemented at the Technical University of Cluj-Napoca (TUCN), creating the research context of this paper and a detailed state of the art regarding outlier detection techniques is made, the second section of the paper presents a brief description of the TUCN pilot locations and the DR-BoB implemented system architecture with its components. The third section introduces the applied baseline evaluation approach, showcasing the implemented outlier detection techniques with a special highlight on the proper tuning parameter values to be applied for each investigated outlier detection technique in case of hourly energy consumption data. Additionally, to test and validate the detected outlier data points, an integrated custom scoring method is presented in section three of this paper. Obtained results after the outlier detection and removal processes was applied are highlighted in section four, along with the baseline consumption curves constructed on the original and the cleared data sets. Final conclusions and comments are outlined in the last section of the article.

## 2. Overview of the Implemented Demand Response System

Regarding the implemented demand response pilot project, the following clarifications have to be made regarding the existing monitoring system:

1. The only form of energy involved in the demand response pilot process is electricity.
2. When the project started, there was only one system for measuring and settling electricity consumption, the one installed by the electricity distribution system operator (DSO). It was combined by three-phase electronic meters, mounted in a semi-direct current and voltage measurement scheme, for currents featuring current reducers and for voltages directly connected to the grid. The meters in each location have the possibility of remote data transmission, but the direct beneficiary of this data is the local electricity distribution system operator (DSO). The technical university came into possession of the measurements by directly requesting this data from the beneficiary. The measured data had a sampling frequency of 1 h.
3. One of the main objectives of the demand response pilot project was to implement a system for monitoring and remote management of electricity consumption in each involved group of buildings. The monitoring [50] and remote management system has a relatively simple architecture, monitoring only a small number of consumers in each location, therefore the equipment is considered to have a proportional impact on the project. This objective could be achieved only after identifying all the electricity consumers, the operating regimes and establishing their importance according to their energy consumption.
4. It is also specified that within the technical university there is only one type of monitoring systems, the one described at point 2, which are also settlement systems in the relation with the local electricity distribution system operator (DSO) and further with the electricity supplier. The only exceptions are the four groups of buildings involved in the demand response pilot project.

**Figure 1:**
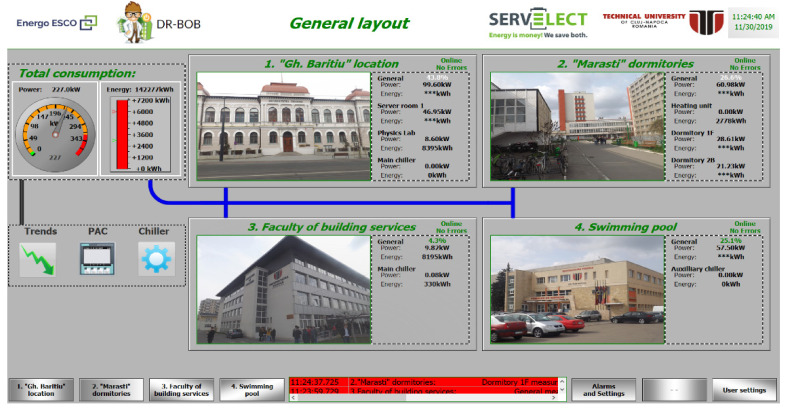
General layout—overview of consumption on the four locations plus aggregate consumption total.

The monitoring system of the energy consumption in the pilot buildings for the demand response project has the following structure:

- Semi-direct mounted meters, with intermediate measurement (current reducers) for current and direct measurement for voltage;
- Communication module between meters and PLC or data totalizer;
- Communication module between the PLC or data totalizer and the computer on which the application is running, the graphical interface;
- Local data server—dashboard, for storing measured data;
- The computer on which the application runs with the graphical interface;
- Monitors mounted in the main access ways in buildings, on which the monitoring system is displayed and where certain elements can be viewed by the occupants of the building. The following print-screen highlights the structure of the implemented monitoring system in each pilot location, with the corresponding real-time consumption:

Figure 1 shows the general view/graphical interface of the monitoring systems that was implemented at four pilot site locations. The related daily consumptions in (kWh) and the absorbed power in (kW) are presented. Also, the total or aggregate electricity consumption is presented [51] for the four pilot groups of buildings:

- The block of buildings from the Faculty of Electrical Engineering;
- The block of buildings from the MarastiStudents Campus;
- The Faculty of Building Services;
- The swimming pool complex.

Through the monitoring system, the four groups of Technical University of Cluj-Napoca (TUCN) pilot buildings were interconnected with other pilot sites in Europe, in this way the demand response events were tested on a complex platform, with a series of integrated utilities and functionalities. The role of this platform was to manage and aggregate all the factors that influence the development of demand response events and all the participating pilot sites in a centralized process of reaching consumption and energy cost reduction targets, power peaks, CO_2_ emissions, etc.

**Figure 2:**
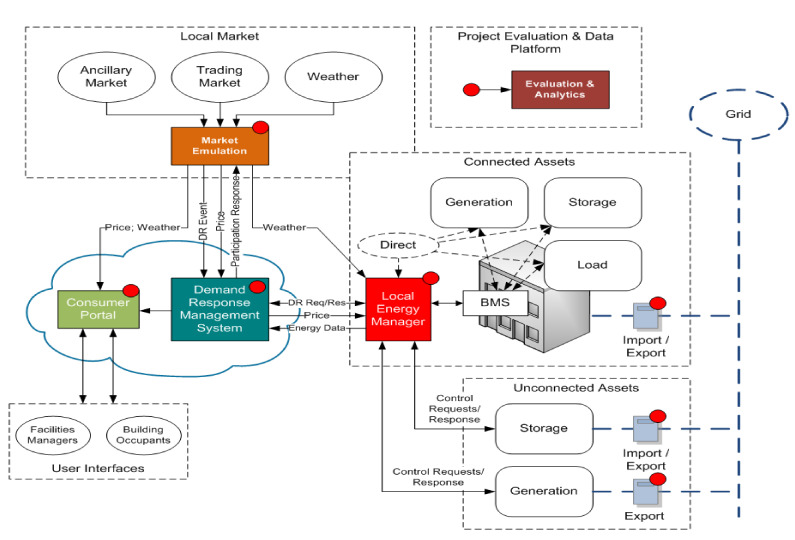
Demand response system overview.

Figure 2 shows an overview of the aggregated system that includes a series of data information and equipment for the centralized management of several pilot sites. As main functionalities and subsystems, the following are highlighted [52]:

1. Market emulation (ME) unifies information from the energy market (from electricity distribution system operator (DSO), electricity transmission system operator (TSO), energy suppliers, aggregators, etc.) and from regional meteorological operators about the factors that are influencing energy consumption and production. It receives notifications and forwards them to the pilot sites for demand response (DR) events and communicates with the local energy manager (LEM) to provide relevant information on weather conditions.
2. Local energy manager (LEM) [53] may take over a range of information (energy consumption, energy production, energy storage) directly from energy consumers or, indirectly through monitoring systems or building management system/building energy management system (BMS/BEMS) implemented at each pilot site. Local energy manager (LEM) has also the role of performing the necessary calculations in order to quantify the results obtained by implementing demand response events, through base line forecasting algorithms and key performance indicators (KPIs) calculation. At the same time, it conveys the obtained results further to demand response management system (DRMS).
3. Demand response management system (DRMS) is an intermediary between local energy manager (LEM) and the market emulator, it facilitates the exchange of information to achieve the predicted events in high performance conditions. Also, through another functionality system called consumer portal (CP), it transmits information regarding the development of events: market notifications, implementation period, equipment that should be involved and obtained results quantified by local energy manager (LEM), involving the Technical University of Cluj-Napoca (TUCN) staff, students, or other stakeholders.
4. Consumer portal (CP) makes all the relevant information about demand response events available to building owners, administrators, and occupants. In short, the consumer portal (CP) is the interface between all market participants in demand response and system functionalities.
5. The electricity network has the role of supplying electricity to the consumers in each building or group of buildings, respectively, it is the element through which the electricity produced and/or stored locally at each location, is injected in the network.

By using the above-presented various interconnected technologies with clear and specific functionalities, the possibility of interaction between energy consumers and facility management teams responsible for demand response programs is created. In addition, the use of advanced predictive control and forecasts—which can provide an accurate and highly detailed view of the operation of the building group and their consumers, in terms of energy consumption and production—influence the various types of behaviors.

## 3. Applied Methods

### 3.1. Baseline Determination

The biggest challenge in evaluating the effectiveness of a demand response (DR) event is to properly determine what would be the real energy consumption in the absence of the event. Hence, to calculate the key performance indicators (KPI’s) [54] for DR events taking place within the four Technical University of Cluj-Napoca (TUCN) blocks of buildings, it was impetuously necessary to identify a baseline consumption level for each day when demand response events took place. The starting point in determining the baseline reference consumption was a database composed of hourly electricity consumption values, starting 2014 until fall of 2019 for each block of buildings within TUCN, included in the pilot project.

The electricity consumption data at hourly level were taken from two main sources, namely: the local electricity distribution system operator (DSO), as settlement meter records for each analyzed block of buildings (for the historical energy consumption between 2014 and 2018) and the energy monitoring system, presented in the previous section (Section 2) and implemented within the demand response pilot project (for the energy consumption data starting from 2018 onwards). Given the different nature of energy consumption in the analyzed buildings at different times of the year, an energy profiling action had to be applied for each block of building separately. The applied energy profiling action determined the average daily consumption schedules (baseline consumptions) for each day of the week separately and for similar days, as occupants’ activity like weekdays and weekends, respectively looking also for activity patterns. Taking into consideration that the analyzed blocks of buildings belong to the Technical University of Cluj-Napoca, their activity patterns mostly correlate to the academic year schedule: teaching semesters, examination periods and student vacations, in other words, there is a link between energy consumption and the social system of the tested buildings [55]. An exception is made in the case of the swimming pool complex, where the activity pattern is correlated to weather conditions (an additional outdoor pool is operated during summer periods) and special sporting events.

To obtain the proper average daily schedule (baseline consumption) for each analyzed block of buildings, a first selection (correction) of the used energy consumption data was made by identification of atypical activity patterns at day level and extracting from the process of baseline consumption curve evaluation. For these corrections we listed the days considered atypical: the beginning of the calendar year, when it is a national holiday, the holiday at the end of the first semester session, the Easter holiday, on the first day of May, the holiday at the end of the second semester session, respectively the summer vacation, when the hourly consumption profile is very different from the days when current activities are carried out, depending on the specifics of each group of buildings. The second correction consisted in eliminating from the baseline evaluation for each day of the week, the energy consumption data related to the demand response (DR) events that were carried out. Especially during the events, but also 2 h before and 2 h after the event, the consumption profile underwent changes, so that they would have had a negative impact in generating the baseline reference level. With all these assumptions taken into consideration the generated baseline consumption curves would be as good as the input energy consumption data used for the study. Consequently, a data cleaning process, applied with outlier detection and data correction, is presented in the next section.

### 3.2. Data Cleaning

The proposed outlier detection techniques for the analysis are: interquartile range (IQR) [56], median absolute deviation (MAD) [57,58], local outlier factor (LOF), and density-based spatial clustering of applications with noise (DBSCAN).

To test the universal data approach of the methods the authors applied the most common input threshold/parameters values from literature. The Interquartile range (IQR) method helps not only in outlier detection but also in predicting the spread [56] of energy consumption, yet it is tight to its mathematical limitation of identifying only the values which are between the tested threshold value. The same can be observed in the mathematical model of the median absolute deviation (MAD) [57,58] where the limitations are given by the threshold values from the median value. The threshold value chosen for Interquartile range (IQR) method testing is 1.5 [59] and for median is 3. The advantage of using the IQR and median absolute deviation (MAD) models is more related with ‘tracking’ and maintaining a permeant control spread that will identify the extreme values for most of the cases. A confirmation of an outlier from both methods should always be taken into consideration for future investigations.

The local outlier factor (LOF) method [60] can achieve good results when the outliers are located in dense region of normal data which means that the accuracy of this method can be reduced when it is exposed to a high volatility data set. In the performed analysis values of *k* equal with 2, 3, 4, 5, 25, and 50 were used. It was determined that the most suitable *k* values for hourly energy consumption data sets would be the 2 and 3 which are the most common in the literature [61], and also the value of 25, which was empirically tested with a good accuracy, compared with the other values. For the density-based spatial clustering of applications with noise (DBSCAN) method, [62,63] the authors used as input parameter 0.5 for epsilon (ε) as a default value, and the minimum number of points equal to 5. For these values, the highest silhouette score [64] was obtained following various testing scenarios, where the authors applied different epsilon (ε) values (from 0.1 to 0.9) and used various minim number of points (5, 10, 20, and 50).

### 3.3. Outlier Verification and Validation

Due to a need of outlier validation, the support function or the “Custom Scoring Method” (CSM) was designed to analyze the output from any outlier detection method and to decide in the context of electrical energy consumption, if the detected anomalous value is a natural energy peak, or abnormal data. The method compares the input (detected outlier values) with the average energy consumption of four data clusters for the same interval of time and similar days (workdays or weekends).

**Figure 3:**
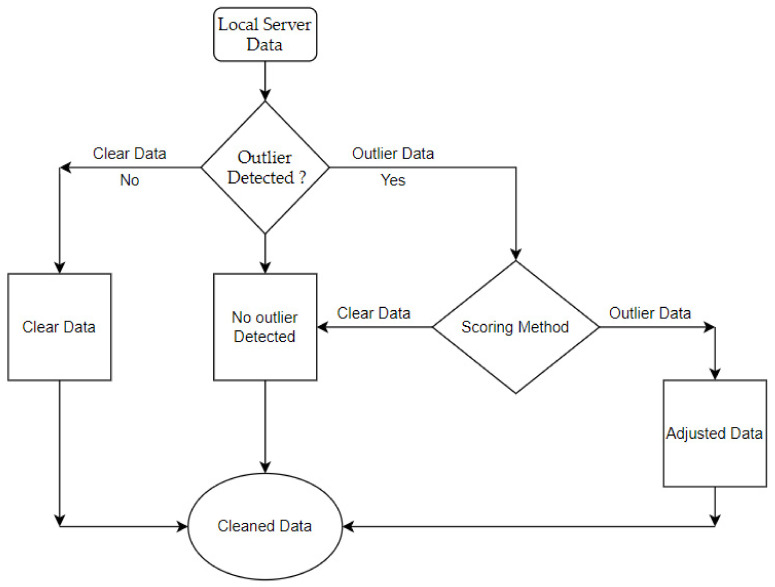
Flowchart/algorithmic diagram of the proposed methodology.

An overview of the applied outlier verification methodology is presented in Figure 3. The data is collected in the local server and then it is analyzed using interquartile range method, median absolute deviation, and density-based spatial clustering of applications with noise, with the parameters presented in the previous section.

To verify and validate the outliers identified by the above presented outlier detection techniques, an outlier ranking system has been developed and implemented. As a first step for each identified outlier data point, four data clusters have been selected from the available historical data sets, consisting of similar energy consumption values for the same hour of the day, as follows:

Cluster A—energy consumption data from the same location for similar days: weekday (Monday to Friday) or weekends (Saturday and Sunday), from the same year, as the analyzed outlier data point (national holidays not included).

Cluster B—energy consumption data from the same location for similar days: weekday (Monday to Friday) or weekends (Saturday and Sunday), same period of the year (2 month period starting from 15th of the previous month to 15th of the next month), the same year as the analyzed outlier data point (national holidays not included).

Cluster C—energy consumption data for similar days: weekdays (Monday to Friday) or weekends (Saturday and Sunday), from the entire available historical data sets, for the same location as the analyzed outlier data point (all years, national holidays not included).

Cluster D—energy consumption data for similar days: weekdays (Monday to Friday) or weekends (Saturday and Sunday), same period of the year (2 month period starting from 15th of the previous month to 15th of the next month) from each year within the entire available historical data sets, for the same location as the analyzed outlier data point (all years, national holidays not included).

The above-mentioned data clusters were selected in order to accurately identify general daily energy consumption patterns (Clusters A and C), specific to each pilot location, respectively each day type and to catch seasonal energy consumption pattern changes (Cluster B and D), but agreeing to not be influenced by changes in buildings infrastructure (replacement of old equipment or acquisition of new equipment, that could significantly change the average energy consumption from one year to another) (Cluster A and B).

As a second step, a score (from 0 to 5) is given for the analyzed outlier data point, according to each of the above presented data clusters, based on how close the corresponding hourly energy consumption value of the outlier is to the central/average energy consumption of the data cluster. For this the average, the minimum and the maximum energy consumption over the closed ***P***% of the data points from a data cluster is evaluated according to Equation (1), where ***P*** could be 75%, 90%, or 60% of all the data points from a cluster:

(1) $$avgW_{P\%}=\frac{1}{N_{P\%}}\cdot \overset{N_{P\%}}{\underset{i=1}{\sum }}W_{i},\;minW_{P\%}=\min\left\{W_{i}\right\},\;maxW_{P\%}=\max\left\{W_{i}\right\},\;i=\bar{1,N_{P\%}}$$

with: $avgW_{P\%}$—the average hourly energy consumption; $minW_{P\%}$—the minimum hourly energy consumption; $maxW_{P\%}$—the maximum hourly energy consumption over the closed ***P***% of the data points from a cluster; $N_{P\%}$—the number of data points from the closed ***P***% range; and $W_{i}$—a hourly energy consumption data value from the specified data range within a cluster.

**Figure 4:**
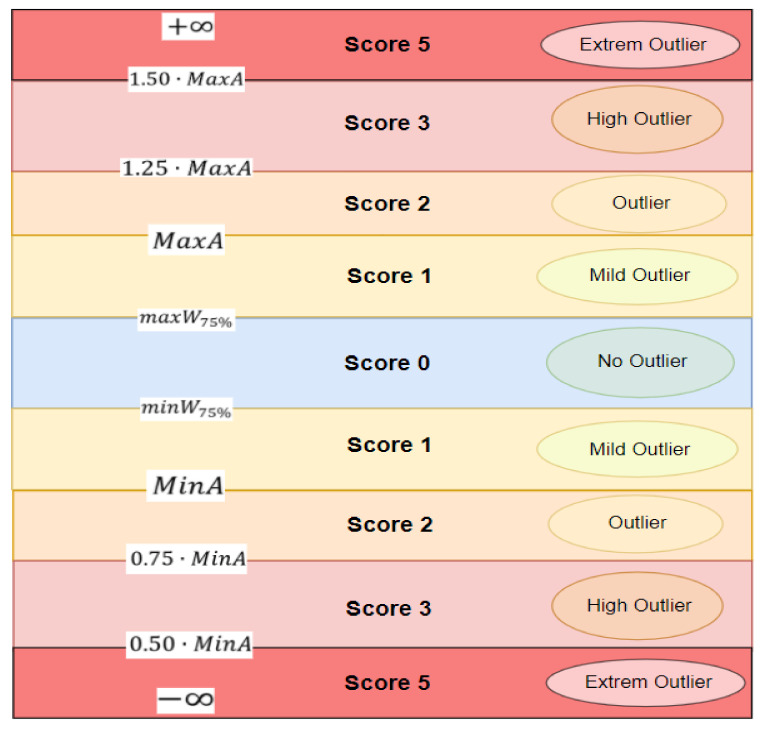
Outlier scoring range over cluster A.

The applied scoring algorithm is mathematically described through (2) to (6) and graphically represented in Figure 4. Namely if the hourly energy consumption corresponding to the analyzed outlier is between the closed 75% range of a specific data cluster then the analyzed data point is not a real outlier and therefor the Score is set to 0, see (2). If the analyzed outlier data point is between the 75% range limit and a restrictive 90% data range limit, then there is a mild outlier and the score is set to 1, see (3). If the outlier hourly energy consumption value exceeds the restrictive 90% data range limit, then we have a real outlier data point and the score is set to 2, see (4). If the outlier data value not only exceeds the restrictive 90% data range limit, but is more than 25% higher or smaller then a high outlier is found and the score is increased to 3, see (5), while if it is more than 50% higher or smaller, an extreme outlier is detected and the score is increased to 5, see (6).

(2) $$\mathrm{IF}\;\;Out_{j}\subset \;\left[minW_{75\%},maxW_{75\%}\right]\;\;\mathrm{THEN}\;\;Score=0$$

(3) $$\mathrm{IF}\;\;Out_{j}\subset \;\left[\left.MinA,minW_{75\%}\right)\right.\;\cup \;\left(\left.maxW_{75\%},MaxA\right]\right.\;\;\mathrm{THEN}\;\;Score=1$$

(4) $$\mathrm{IF}\;\;Out_{j}\subset \;\left[\left.0.75\cdot MinA,MinA\right)\right.\;\cup \;\left(\left.MaxA,1.25\cdot MaxA\right]\right.\;\;\mathrm{THEN}\;\;Score=2$$

(5) $$\mathrm{IF}\;\;Out_{j}\subset \;\left[\left.0.50\cdot MinA,0.75\cdot MinA\right)\right.\;\cup \;\left(\left.1.25\cdot MaxA,1.50\cdot MaxA\right]\right.\;\;\mathrm{THEN}\;\;Score=3\;$$

(6) $$\mathrm{IF}\;\;Out_{j}\subset \;\left(-\infty ,0.50\cdot MinA\right)\;\cup \;\left(1.50\cdot MaxA,+\infty \right)\;\;\mathrm{THEN}\;\;Score=5$$

with $Out_{j}$—the hourly energy consumption value of the *j*th analyzed outlier data point and

(7) $$minA=\max\left\{minW_{90\%},minW_{75\%}+\;\left(minW_{75\%}-minW_{60\%}\right),minW_{75\%}\cdot \frac{minW_{60\%}}{minW_{75\%}}\right\}$$

(8) $$maxA=\min\left\{maxW_{90\%},maxW_{75\%}+\;\left(maxW_{75\%}-maxW_{60\%}\right),maxW_{75\%}\cdot \frac{maxW_{75\%}}{maxW_{60\%}}\right\}$$

Based on the scores obtained for data clusters A and B, a first mark is evaluated for each analyzed outlier data point as a weighted average value of the two scores, according to Equation (9), while a second mark is computed as a weighted average value of the scores obtained for data clusters C and D, according to Equation (10)

(9) $$Mark1=\frac{Score_{A}+1.5\cdot Score_{B}}{2}$$

(10) $$Mark2=\frac{1.5\cdot Score_{C}+2\cdot Score_{D}}{2}$$

(11) Rank=Mark1+Mark2

The final rank is obtained as the sum of the two marks computed for the analyzed outlier data point, see Equation (11). If the final rank is lower than 3, then the data point is not considered a valid outlier. The adjustment of the valid outliers was conducted using one of the most popular outlier detection techniques in literature, that is the shape-preserving piecewise cubic spline interpolation [65].

## 4. Results and Observations

### 4.1. Data Cleaning

The need for interconnectivity and balance between production and consumption of electrical energy is an “impetuous agreement” of clean data [49]. Any forecasting process [66,67], or statistical model can be jeopardized by the absence of a cleaned data set.

#### 4.1.1. Swimming Pool Complex

It has been observed that from a common ground of 413 outliers, only 322 were validated through the system. The same process was conducted over the combined data of DBSCAN results and 95.2% of the observation were validated as real outliers. Because the LOF method had a larger number of detected issues, it was decided to run the k = 2 and k = 3 detected outliers independently. For both of the local outlier factor (LOF) computations, the results lacked accuracy, having only 755 valid outliers out of 3512 detected for k = 2 and only 292 outliers out of 1628 for k = 3, as shown in Table 1. 

**Figure 5:**
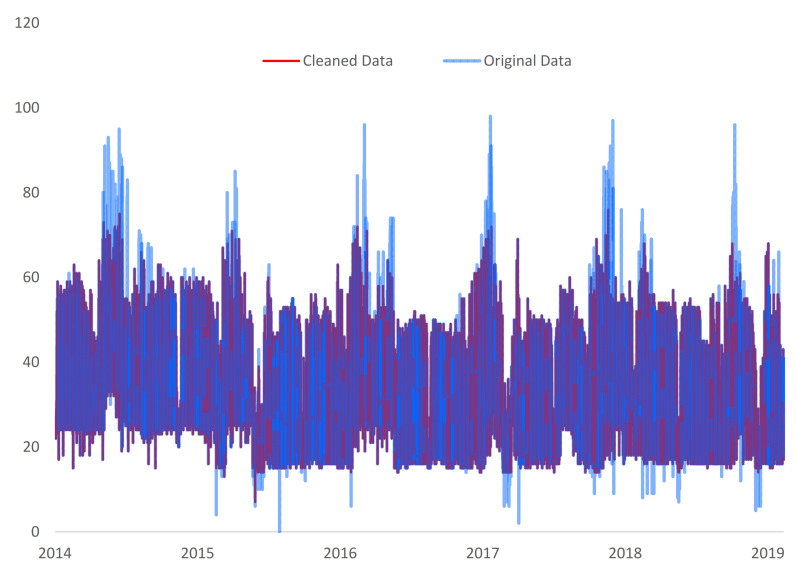
Original and cleaned data for swimming pool complex location.

After the scoring process, the valid outputs were analyzed in one database. It turned out that from all the methods available, there is a total of 1468 unique valid outliers. Some of the methods validated the same data point as an anomaly. All methods detected 23 common data points, 63 were detected by three of them, and 416 by any two methods that had a common value (see Table 2). Thus, a total of 502 common outliers were identified. Despite that, due to the low percentage of the already filtered outliers through the implemented scoring method, compared with all the data set, it was decided to adjust all the 1468 unique outliers (see Figure 5）。

#### 4.1.2. Faculty of Electrical Engineering

**Figure 6:**
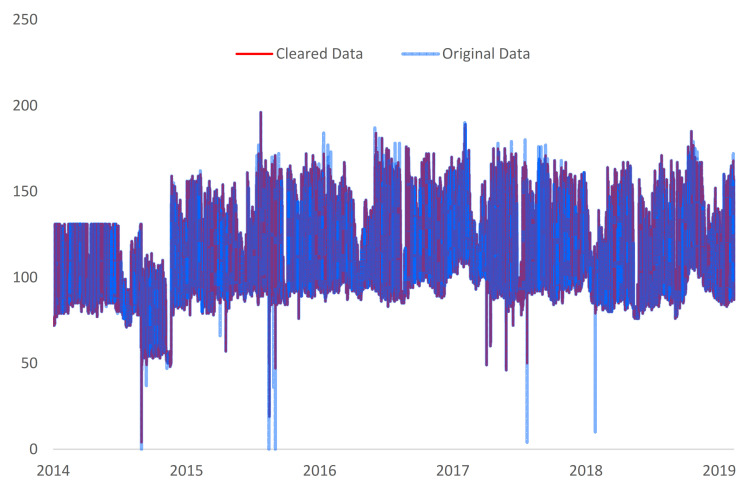
Original and cleaned data for Faculty of Electrical Engineering location.

To confirm and to conclude the previous observations, the data collected from Faculty of Electrical Engineering, Faculty of Building Services and Marasti Students Campus were analyzed using the proposed methodology. In the first iteration, the data from the Faculty of Electrical Engineering block of buildings was first analyzed using interquartile range (IQR) and median absolute deviation (MAD) algorithms on each year, to extract yearly outliers for each year individually and then the same process was executed on all the data set. There are multiple ways data can be analyzed, because the authors wanted to highlight the most obvious outliers, the intersection of the two processes had been made, the result being represented by the interquartile range (IQR)/comb and median absolute deviation (MAD)/comb. It was concluded that the interquartile range (IQR) method detected that 1.4% of all data is represented by outliers and for median absolute deviation (MAD) 3.7%. The process was also conducted on all the data and the same outlier percentage were obtained for k = 2 and k = 3, and less than 1% for k = 25. In the case of the density-based spatial clustering of applications with noise (DBSCAN) process, the intersection between two different density-based processes had been made: one was based on the energy consumption value and hour (DBSCAN 1) and the second one was based on the energy consumption value and the day of the week (DBSCAN 2). The testing was also conducted on yearly data and on the entire data set, respectively. Given that no intersection elements were found between the two processes, the combination (reunion) of the results obtained with these two approaches has been applied. The data in Table 3 shows that in total, the DBSCAN method detected 2.2% of the data to be an outlier. With the aim of understanding the validity of the tested methods the implemented scoring method was compiled over the outlier outputs. Given that the interquartile range (IQR) and median absolute deviation (MAD) are both ‘spread control’ based methods, the intersection of these two was used as input for the outlier scoring/validation. Based on this process, 966 unique outliers were adjusted from the data set (see Figure 6).

#### 4.1.3. Faculty of Building Services

**Figure 7:**
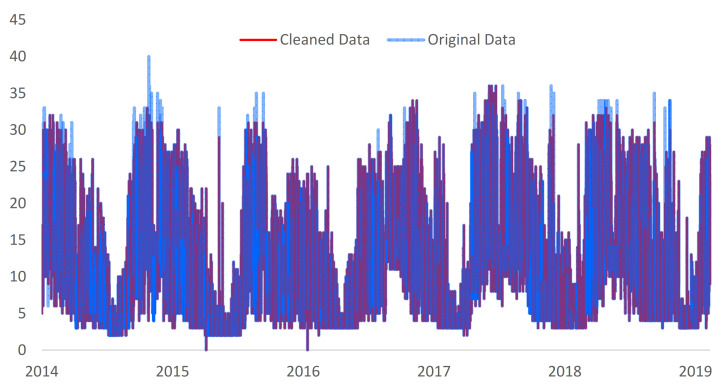
Original and cleaned data for the Faculty of Building Services location.

For the data collected from Faculty of Building Services the same test was applied using interquartile range (IQR) and median absolute deviation (MAD) process. It was observed that from all the data, IQR detected 2% of the data as outliers and 3% for MAD (see Table 4). In the case of the local outlier factor (LOF) process, 2.2% of the data were outliers for k = 2, 0.8% for k = 3, and 0.09% for k = 25 respectively for yearly data. For all the process, all the data was tested and we obtain 6.8% of the data as outlier based on k = 2 and 0.8% for the k = 3, the algorithm found only one outlier. Compared with other methods for the density-based spatial clustering of applications with noise (DBSCAN) only 0.4% of the data were marked as outlier. Even if the total number of anomalous data is low, the method indicated a silhouette score equal with 0.99 for both DBSCAN 1 and DBSCAN 2. The implemented scoring method was used to validate the results from Table 4 and Figure 7.

#### 4.1.4. Marasti Students Campus

**Figure 8:**
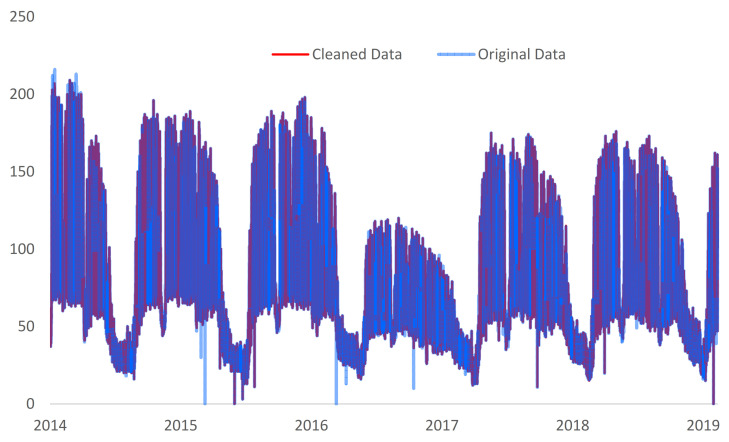
Original and cleaned data for Marasti Students Campus location.

Regarding Marasti Students Campus data, abnormal behaviors were observed in the interquartile range (IQR) and median absolute deviation (MAD) algorithms. For both methods and approaches zero outliers were detected for most of the tested years even if a visual data interpretation suggests otherwise. Continuing the analysis on the local outlier factor (LOF) process, 5% of the data was detected as outliers for k = 2, 1.5% for k = 3, and 0.8% for k = 25 respectively. Running the same exercise for the entire historical energy consumption data set, the same proportions of outliers were found k = 2 and k = 3, while for k = 25 only 0.02% of the data was detected as outliers (see Table 5). In case of the density-based spatial clustering of applications with noise (DBSCAN), when the energy consumption and hour values were used for the clustering process (DBSCAN 1), an average of 52% of the data was marked as outlier, but no intersection between the yearly data sets and the entire data sets results was found. In the second part of the analysis, the opposite was observed for DBSCAN 2 (when the energy consumption and the day of the week were considered for the clustering process), only 2.2% of the data were marked as outliers (see Table 5 and Figure 8). It is worth mentioning that for this experiment, there were also no outlier intersection between the yearly data and total data approach. Moreover, the same could be noticed between DBSCAN 1 and DBSCAN 2 results. Therefore, to understand the huge consistency gap that occurred between IQR, MAD, LOF, and DBSCAN processes for this data set, the standard deviation (SD) of all the data sets/pilot locations were calculated and compared.

The evaluation of the data sets standard deviation (SD) has revealed much higher SD values for Marasti Students Campus and Faculty of Electrical Engineering locations (38.98 and 22.74 respectively) with respect to the Swimming Complex and Faculty of Building Services locations (10.35 and 6.44 respectively). The SD value from Marasti Students Campus data set indicates a random behavior in energy consumption, which is almost double compared to the Faculty of Electrical Engineering data and significantly higher compared to the other locations.

### 4.2. Impact on Baseline Construction

Given that the efficiency of the load reduction (demand response effect) can be quantified only from the baseline curve, [68] an investigation of the impact of the cleaning methods over this methodology is mandatory. The aim of this study is to understand if integration of cleaning methods algorithms in a complex demand response automated platform can improve the baseline accuracy.

Due the fact that all of the tested locations are defined by different social-energetic behavior [60], the baseline analysis was conducted independently for each of the investigated location, in accordance to their specific energy profile.

#### 4.2.1. Swimming Pool Complex

**Figure 9:**
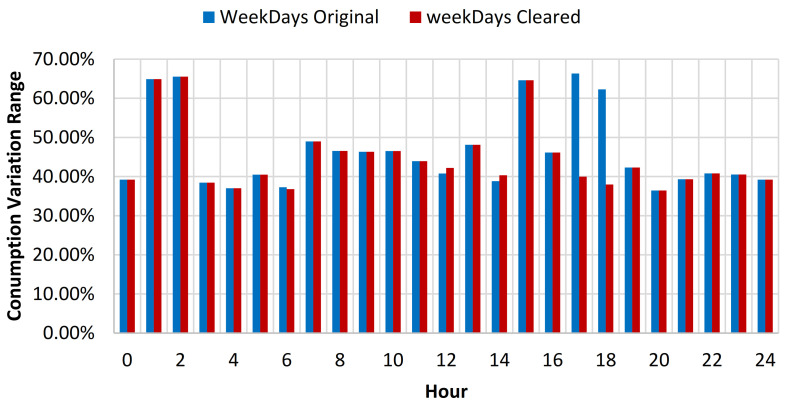
Energy consumption variation range compared to hourly average weekdays consumption for original and cleared data sets.

**Figure 10:**
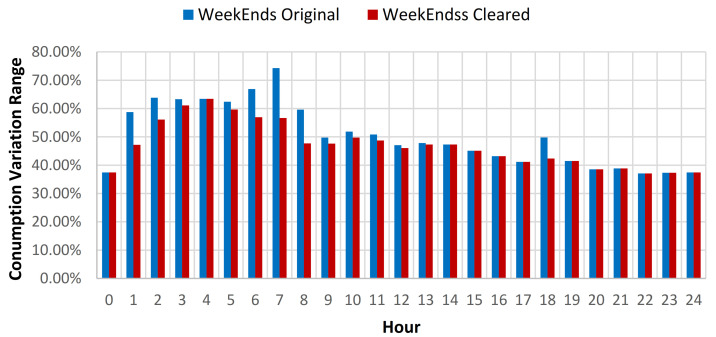
Energy consumption variation range compared to hourly average weekends consumption for original and cleared data sets.

For the swimming pool complex location where throughout the summer period (June 1–August 31) an additional open air swimming pool is open for public usage, the consumption baseline investigation was considered using data collected throughout the full year, the summer period, and the rest of the year energy consumption data. Based on the results obtained from the full year energy consumption baseline analysis, it has been concluded that after the cleaning process, the yearly standard deviation of the baseline was reduced on average with 0.52% for all days from the data set, for weekdays with 0.43% and for weekends with 0.75% (see Table 6, Figure 9 and Figure 10).

**Figure 11:**
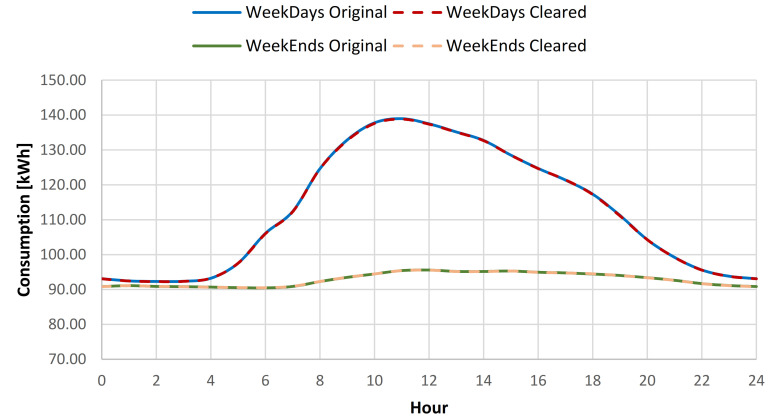
Daily consumption baseline for the original and cleared data sets.

For the summer period consumption, a reduction of the standard deviation (SD) of 0.36% for all the data, 0.41% for weekdays data, and 0.23% for weekends, was obtained (see Table 7). For the rest of the year data, an increase to 0.63% of the SD reduction was observed for all the data, 0.41% for weekdays data and 1.2% for weekend data (see Table 8). In order to provide better understanding of the impact of the data cleaning over the consumption profile, a plot was created for both weekdays and weekends, for both original and cleared full year data sets (see Figure 11).

#### 4.2.2. Faculty of Electrical Engineering

The energy profile of the Faculty of Electrical Engineering is more dynamic during the semesters; therefore the analysis was conducted for full year data, 1st semester and 2nd semester data, separately. The same approach was applied both for the Faculty of Building Services as for Marasti Students Campus locations.

**Figure 12:**
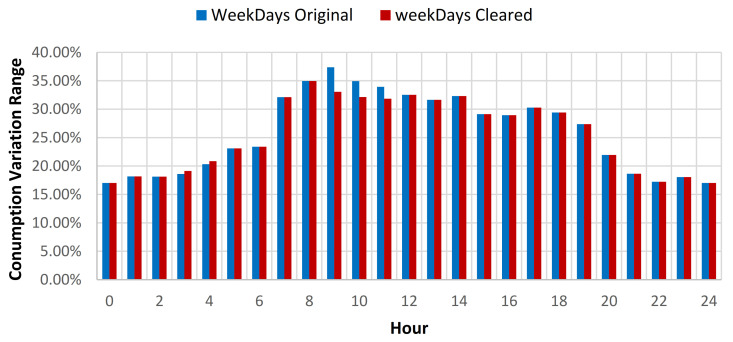
Energy consumption variation range compared to hourly average weekdays consumption for original and cleared data sets.

**Figure 13:**
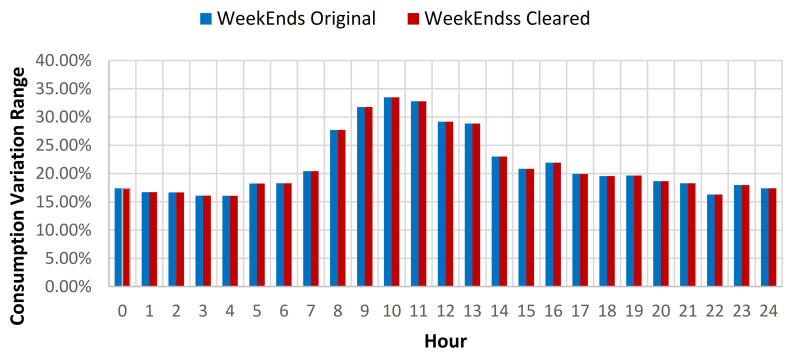
Energy consumption variation range compared to hourly average weekends consumption for original and cleared data sets.

**Figure 14:**
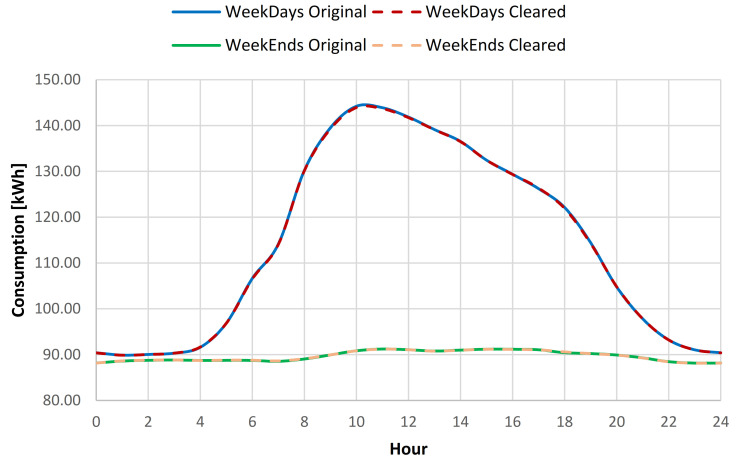
Daily consumption baseline for the original and cleared data sets.

The results showcased that for the baseline curve of the full year data set, an average standard deviation (SD) reduction of 0.79% is obtained for all days, 0.45% for weekdays, and 1.6% for weekends (see Table 9, Figure 12 and Figure 13). The consumption baseline for original and cleared data sets are presented in Figure 14. For the 1st semester (see Table 10) an average SD reduction of 1.09% for all days is recorded, 0.51% for weekdays and 2.57% for weekends, while for the 2nd semester (see Table 11) 0.24% for all days, 0.26% for weekdays and 0.19% for weekend days.

#### 4.2.3. Faculty of Building Services

**Figure 15:**
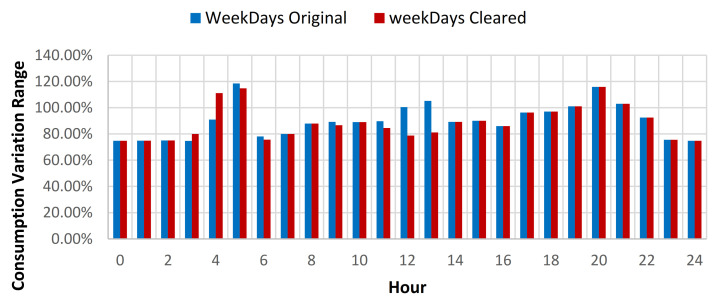
Energy consumption variation range compared to hourly average weekdays consumption for original and cleared data sets.

**Figure 16:**
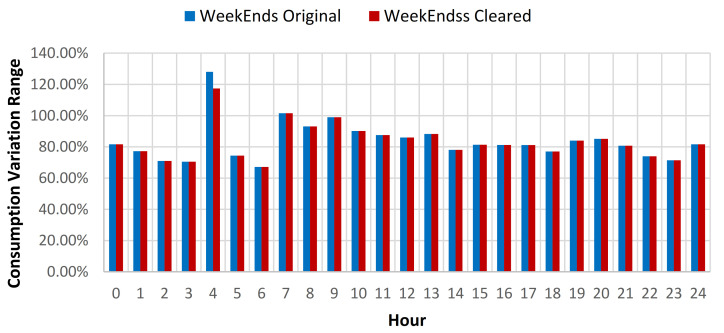
Energy consumption variation range compared to hourly average weekends consumption for original and cleared data sets.

At the Faculty of Building Services location, the results showcased that due to the data cleaning process, a standard deviation (SD) reduction of 0.22% for all days, 0.24% for weekdays and 0.17% for weekend was recorded over the entire historical data sets (see Table 12, Figure 15 and Figure 16).

**Figure 17:**
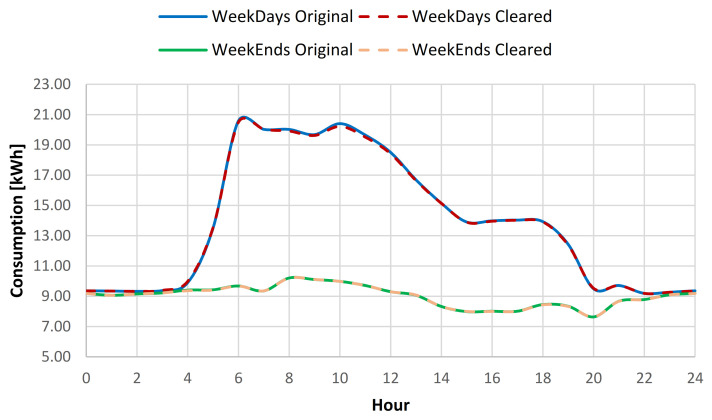
Daily consumption baseline for the original and cleared data sets.

It had been observed that for the 1st semester, the standard deviation (SD) reduction increased to 0.31% for weekdays, 0.27% for all the data, and 0.18% for weekends (see Table 13). For the 2nd semester, a 0.16% average SD reduction for all the days, 0.17% for weekdays and 0.13% for the weekend days was noticed (see Table 14). The daily consumption baseline for original and cleared data sets are showcased in Figure 17.

#### 4.2.4. Marasti Students Campus

**Figure 18:**
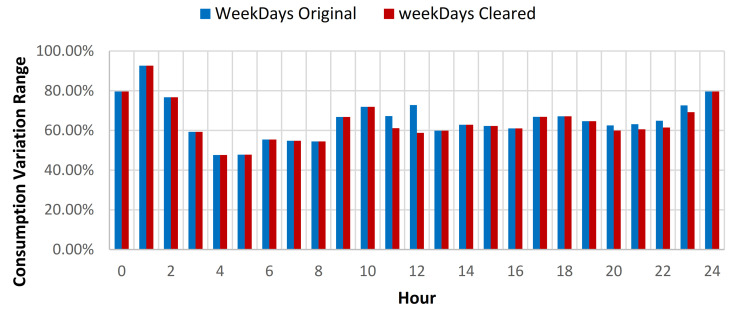
Energy consumption variation range compared to hourly average weekdays consumption for original and cleared data sets.

**Figure 19:**
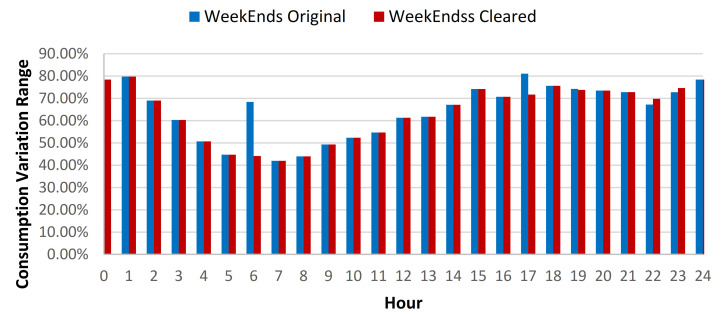
Energy consumption variation range compared to hourly average weekend consumption for original and cleared data sets.

Even if the Marasti Students Campus data is the most volatile set and there were issues encountered for the DBSCAN algorithm during the outlier detection process, after the adjustment of those detected and validated, an average reduction of standard deviation for the full year data of 0.66% for all days, 0.61% for weekdays, and 0.78% for the weekend days was recorded (see Table 15, Figure 18 and Figure 19).

**Figure 20:**
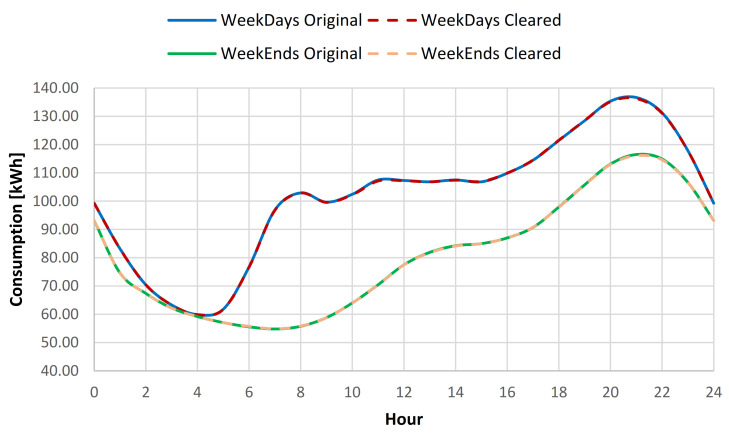
Daily consumption baseline for the original and cleared data sets.

For the 1st semester, an average standard deviation (SD) reduction of 0.65% for all the data, 0.74% for weekdays, and 0.44% for the weekend days respectively was noticed (see Table 16). In the 2nd semester, a SD decrease of 0.67% for all days, 0.59% for weekdays, and 0.87% for weekend days was computed (see Table 17). The daily consumption baseline for original and cleared data is highlighted in Figure 20.

## 5. Conclusions

During the DR-BOB “Demand Response in Blocks of Buildings” project, the energy demand from 12 buildings at four different locations was monitored, 36 Demand Response events were successfully implemented during an evaluation period of one year. In order to improve the quality of baseline data and key performance indicators (KPI) evaluation, a data cleaning process was proposed (interquartile range, median absolute deviation, local outlier factor, density-based spatial clustering of applications with noise, intelligent scoring method). The numerical results showcased that even in case of different energy profiles, the cleaned data was reduced in all the cases, the standard deviation of the baseline with an average of 0.41%, which means that the nature of the data is not affected by the removal of outliers and we also gain more accuracy in baseline. More than that, this highlights the fact that a cleaning process in data energy, before any qualitative or quantitative process, can significantly improve the quality of the results. It is also important to mention that in addition to the proposed outlier detection techniques a custom integrated function was created in order to differentiate between natural energy peaks and real outliers.

For the demand response project implemented at the level of the Technical University of Cluj-Napoca’s block of buildings, in some cases, it was impossible to compute the required KPIs (power reduction, energy savings, cost savings, CO_2_ emissions reduction) without the data cleaning process or the resulted values which were far higher (>50%) and not realistic. This is why, in real case business models, in order to rely upon the demand response in blocks of buildings (DR-BoB) demo pilot, it is crucial to use outlier removal techniques. The presented improved baseline data cleaning process will facilitate benchmarking and validating the integration of new incorporated RES technologies [69] and their associated established KPIs in the RE-COGNITION “REnewable COGeneration and storage techNologies IntegraTIon for energy autONomous buildings” project.

## Figures and Tables

**Table 1:** Number of valid/invalid outliers detected using the implemented scoring method for swimming pool complex.

Applied Method	Total Outliers	Valid Data Points	%	Invalid Data Points	%
IQR/Median	413	322	78	91	22
DBSCAN	728	693	95.2	35	4.8
LOF K2	3512	755	21.5	2757	78.5
LOF K3	1628	292	17.9	1336	82.1

**Table 2:** Methods overlap.

Method	Unique	Overlap	Common Outliers
IQR/Median	72	4 Methods	23
DB SCAN	393	3 Methods	63
LOF K2	458	2 Methods	416
LOF K3	43	Total	1468

**Table 3:** Number of valid/invalid outliers detected using the implemented scoring method for Faculty of Electrical Engineering location.

Method	Total Outliers	Valid (%)	Invalid (%)
IQR/MAD	249	46.18	53.81
LOF K = 2	2608	22.17	77.82
LOF K = 3	1412	21.33	78.66
LOF K = 25	18	0	100
DBSCAN	149	17.67	82.32

**Table 4:** Number of valid/invalid outliers detected using the implemented scoring method for Faculty of Building Services location.

Method	Total Outliers	Valid (%)	Invalid (%)
IQR/MAD	227	50.2	49.7
LOF K = 2	3513	20.67	79.3
LOF K = 3	424	17.49	82.5
LOF K = 25	1	0	100
DBSCAN	35	28.57	71.42

**Table 5:** Number of valid/invalid outliers detected using the implemented scoring method for Marasti Students Campus location.

Method	Total Outliers	Valid (%)	Invalid (%)
IQR/MAD	2885	20.24	79.75
LOF K = 2	2558	17.87	82.12
LOF K = 3	743	20.62	79.38
LOF K = 25	9	0	100
DBSCAN	-	-	-

**Table 6:** Full year baseline for original and cleared data.

Day Type	Total Data Sets	Affected Data Sets	Avg. Std. Dev. Reduction	Max Std. Dev. Reduction	Std. Dev. Original Data	Std. Dev. Cleared Data
Monday	282	62	0.63833339	4.57329690	32.5654024	27.9921055
Tuesday	291	83	0.21108976	2.53577735	30.6688720	28.1330946
Wednesday	291	48	0.26101710	1.88609507	24.5919967	22.7059016
Thursday	287	31	0.47489543	9.81717526	20.8840831	11.0669078
Friday	291	20	0.57262822	6.99005974	33.6994119	26.7093521
Saturday	292	13	1.11490524	8.73879107	12.7415075	4.00271646
Sunday	285	244	0.39262403	9.81717526	20.8840831	11.0669078

**Table 7:** Summer baseline for original and cleared data.

Day Type	Total Data Sets	Affected Data Sets	Avg. Std. Dev. Reduction	Max Std. Dev. Reduction	Std. Dev. Original Data	Std. Dev. Cleared Data
Monday	90	14	0.25722712	1.38143413	22.2708947	20.8894606
Tuesday	92	14	0.15995801	0.72414204	21.4514956	20.7273536
Wednesday	92	10	0.31845359	1.14025074	24.9902879	23.8500372
Thursday	88	12	1.07113351	9.81717526	20.8840831	11.0669078
Friday	92	7	0.26361354	0.85073642	22.0690205	21.2182840
Saturday	92	6	0.05318119	0.09530513	9.24769849	9.15239336
Sunday	91	57	0.41621096	9.81717526	20.8840831	11.0669078

**Table 8:** Rest of the year baseline for original and cleared data.

Day Type	Total Data Sets	Affected Data Sets	Avg. Std. Dev. Reduction	Max Std. Dev. Reduction	Std. Dev. Original Data	Std. Dev. Cleared Data
Monday	192	48	0.74948938	4.57329690	32.5654024	27.9921055
Tuesday	199	69	0.22146432	2.53577735	30.6688720	28.1330946
Wednesday	199	38	0.24590224	1.88609507	24.5919967	22.7059016
Thursday	199	19	0.09832402	0.33991601	24.1315461	23.7916301
Friday	199	13	0.73902074	6.99005974	33.6994119	26.7093521
Saturday	200	7	2.02495443	8.73879107	12.7415075	4.00271646
Sunday	194	187	0.38543444	6.99005974	33.6994119	26.7093521

**Table 9:** Full year baseline for original and cleared data sets.

Day Type	Total Data Sets	Affected Data Sets	Avg. Std. Dev. Reduction	Max Std. Dev. Reduction	Std. Dev. Original Data	Std. Dev. Cleared Data
Monday	155	45	0.80005163	4.57329690	32.5654024	27.9921055
Tuesday	159	57	0.24138839	2.53577735	30.6688720	28.1330946
Wednesday	154	32	0.22393069	1.88609507	24.5919967	22.7059016
Thursday	153	17	0.10343966	0.33991601	24.1315461	23.7916301
Friday	152	10	0.92944792	6.99005974	33.6994119	26.7093521
Saturday	148	5	2.82392383	8.73879107	12.7415075	4.00271646
Sunday	154	161	0.42223725	6.99005974	33.6994119	26.7093521

**Table 10:** Semester I baseline for original and cleared data sets.

Day Type	Total Data Sets	Affected Data Sets	Avg. Std. Dev. Reduction	Max Std. Dev. Reduction	Std. Dev. Original Data	Std. Dev. Cleared Data
Monday	76	29	0.93424518	4.57329690	32.5654024	27.9921055
Tuesday	75	36	0.23101876	2.53577735	30.6688720	28.1330946
Wednesday	72	18	0.11072513	0.75340086	25.6966698	24.9432689
Thursday	71	8	0.13420857	0.33991601	24.1315461	23.7916301
Friday	70	8	1.14611244	6.99005974	33.6994119	26.7093521
Saturday	67	3	4.65951546	8.73879107	12.7415075	4.00271646
Sunday	75	99	0.48126673	6.99005974	33.6994119	26.7093521

**Table 11:** Semester II baseline for original and cleared data sets.

Day Type	Total Data Sets	Affected Data Sets	Avg. Std. Dev. Reduction	Max Std. Dev. Reduction	Std. Dev. Original Data	Std. Dev. Cleared Data
Monday	79	16	0.55682583	3.56489776	30.7131421	27.1482444
Tuesday	84	21	0.25916489	1.41308048	30.9749782	29.5618977
Wednesday	82	14	0.36948069	1.88609507	24.5919967	22.7059016
Thursday	82	9	0.07608952	0.17423679	19.6080710	19.4338342
Friday	82	2	0.06278983	0.08220021	20.8347101	20.7525098
Saturday	81	2	0.07053638	0.07194907	7.53926917	7.46732010
Sunday	79	62	0.32798050	3.56489776	30.7131421	27.1482444

**Table 12:** Full year baseline for original and cleared data sets.

Day Type	Total Data Sets	Affected Data Sets	Avg. Std. Dev. Reduction	Max Std. Dev. Reduction	Std. Dev. Original Data	Std. Dev. Cleared Data
Monday	155	39	0.39033099	2.44632664	8.89512160	6.44879496
Tuesday	159	47	0.13894226	0.67964205	6.57178863	5.89214657
Wednesday	154	33	0.38874836	2.68175466	7.56888654	4.88713187
Thursday	153	35	0.22721360	1.71052435	4.26287478	2.55235043
Friday	152	29	0.09221256	0.78275816	6.27567191	5.49291374
Saturday	148	12	0.09477804	0.18552878	4.61860603	4.43307725
Sunday	154	183	0.24704115	2.68175466	7.56888654	4.88713187

**Table 13:** Semester I baseline for original and cleared data sets.

Day Type	Total Data Sets	Affected Data Sets	Avg. Std. Dev. Reduction	Max Std. Dev. Reduction	Std. Dev. Original Data	Std. Dev. Cleared Data
Monday	76	25	0.42311530	2.44632664	8.89512160	6.44879496
Tuesday	75	31	0.15084977	0.67964205	6.57178863	5.89214657
Wednesday	72	17	0.57812764	2.68175466	7.56888654	4.88713187
Thursday	71	18	0.29103627	1.71052435	4.26287478	2.55235043
Friday	70	14	0.12518671	0.78275816	6.27567191	5.49291374
Saturday	67	3	0.07011142	0.08360126	4.54904831	4.46544705
Sunday	75	105	0.30546345	2.68175466	7.56888654	4.88713187

**Table 14:** Semester II baseline for original and cleared data sets.

Day Type	Total Data Sets	Affected Data Sets	Avg. Std. Dev. Reduction	Max Std. Dev. Reduction	Std. Dev. Original Data	Std. Dev. Cleared Data
Monday	79	14	0.33178759	1.81944735	7.27264081	5.45319345
Tuesday	84	16	0.11587146	0.48155030	8.68271229	8.20116198
Wednesday	82	16	0.18753288	1.00132027	5.54020221	4.53888194
Thursday	82	17	0.15963666	0.88795918	5.34040518	4.45244599
Friday	82	15	0.06143669	0.20928486	1.49394914	1.28466428
Saturday	81	9	0.10300024	0.18552878	4.61860603	4.43307725
Sunday	79	78	0.16839576	1.81944735	7.27264081	5.45319345

**Table 15:** Full year baseline for original and cleared data sets.

Day Type	Total Data Sets	Affected Data Sets	Avg. Std. Dev. Reduction	Max Std. Dev. Reduction	Std. Dev. Original Data	>Std. Dev. Cleared Data
Monday	155	25	0.74834470	4.47590704	33.5377788	29.0618717
Tuesday	159	29	0.68563156	3.28522062	8.52096731	5.23574669
Wednesday	154	25	0.61135013	2.17225386	41.1817287	39.0094748
Thursday	153	25	0.40902660	1.66545057	17.0837133	15.4182627
Friday	152	33	0.62982026	3.23430881	31.4490328	28.2147240
Saturday	148	16	0.41804507	0.86318795	9.60826938	8.74508143
Sunday	154	25	1.14515467	5.19967463	33.1356456	27.9359710

**Table 16:** Semester I baseline for original and cleared data sets.

Day Type	Total Data Sets	Affected Data Sets	Avg. Std. Dev. Reduction	Max Std. Dev. Reduction	Std. Dev. Original Data	Std. Dev. Cleared Data
Monday	76	4	0.71235655	1.26626377	41.2124005	39.9461367
Tuesday	75	3	0.98377012	1.96320590	17.2800882	15.3168823
Wednesday	72	3	0.75784598	1.22024600	43.4315845	42.2113385
Thursday	71	5	0.55739444	1.66545057	17.0837133	15.4182627
Friday	70	8	0.69355372	3.23430881	31.4490328	28.2147240
Saturday	67	6	0.50194908	0.86318795	9.60826938	8.74508143
Sunday	75	6	0.38892542	0.92951641	26.9161001	25.9865837

**Table 17:** Semester II baseline for original and cleared data sets.

Day Type	Total Data Sets	Affected Data Sets	Avg. Std. Dev. Reduction	Max Std. Dev. Reduction	Std. Dev. Original Data	Std. Dev. Cleared Data
Monday	79	21	0.75519958	4.47590704	33.5377788	29.0618717
Tuesday	84	26	0.65123095	3.28522062	8.52096731	5.23574669
Wednesday	82	22	0.59137342	2.17225386	41.1817287	39.0094748
Thursday	82	20	0.37193464	1.36790660	13.4076957	12.0397891
Friday	82	25	0.60942555	1.62250577	17.023428	15.4009222
Saturday	81	10	0.36770266	0.71869533	2.71869533	2
Sunday	79	19	1.38396391	5.19967463	33.1356456	27.9359710

## Data Availability

The data presented in this study could be available through the request from the corresponding author. The data are not publicly available due to privacy policies of the Technical University of Cluj-Napoca.
